# Dual inhibition of FAS and HAS2/3 by 4-MU in Realgar-Coptis chinensis unveils a metabolic checkpoint for liver cancer therapy

**DOI:** 10.1007/s13659-025-00540-9

**Published:** 2025-08-21

**Authors:** Songtao Wu, Yingying Wang, Denghui Deng, Guohua Zheng, Hanxiang Mei, Cong Wang, Xiang Zheng, Chun Gui, Fei Liao, Meixian Xiang

**Affiliations:** 1 Hubei Key Laboratory of Resources and Chemistry of Chinese Medicine, School of Pharmacy, Hubei University of Chinese Medicine, Wuhan, 430065 China; 2 School of Pharmacy, Hubei University of Chinese Medicine, Wuhan, 430065 China; 3 Hubei Shizhen Laboratory, Wuhan, 430061 China; 4 Department of Gastroenterology, Wuhan University Renmin Hospital, Wuhan, 430060 China; 5 School of Pharmaceutical Sciences, South-Central Minzu University, Wuhan, 430074 China

**Keywords:** 4-MU, Liver cancer, Cancer, Lipid metabolism, MST

## Abstract

Modern pharmacology has found that both Realgar and *Coptis chinensis* can induce apoptosis in tumor cells, and traditional Chinese medicine theory suggests the possibility of combining the two, however, the specific mechanisms involved have not been elucidated. This study investigated the therapeutic mechanism of the Realgar-Coptis chinensis drug pair (RCCD) against hepatocellular carcinoma (HCC) by identifying its key active compounds and targets. Through integrated LC–MS analysis, transcriptomics, network pharmacology, and bioinformatics, we identified the mechanism of action, key bioactive compounds, and core targets. Molecular docking, molecular dynamics simulations, and microscale thermophoresis (MST) validated the binding affinity between key compounds and core targets. TIMER2.0 database was used to analyze the relationship between the core targets and HCC. H22 tumor xenograft mouse model and immunohistochemistry and pathology analyses were performed to validate the antitumor efficacy of the active compounds. RCCD has a high degree of selectivity of lipid metabolism pathway, 4-Methylumbelliferone (4-MU) was the key active compound with strong binding activity to the core target fatty acid synthase (FAS), and 4-MU down-regulated the expression of FASN in tumor tissues and induced apoptosis in HCC cells. In addition, as a hyaluronan synthase (HAS2/3) inhibitor, 4-MU interfered with the HA-dependent tumor microenvironment and fibrosis process by inhibiting HAS2/3. Thus, 4-MU may inhibit tumor progression by inhibiting FAS and HAS2/3. 4-MU extracted from RCCD exerts anti-HCC effects by modulating the activities of FAS and HAS2/3, thereby reprogramming lipid metabolism and regulating hyaluronan synthesis.

**Graphical Abstract:**

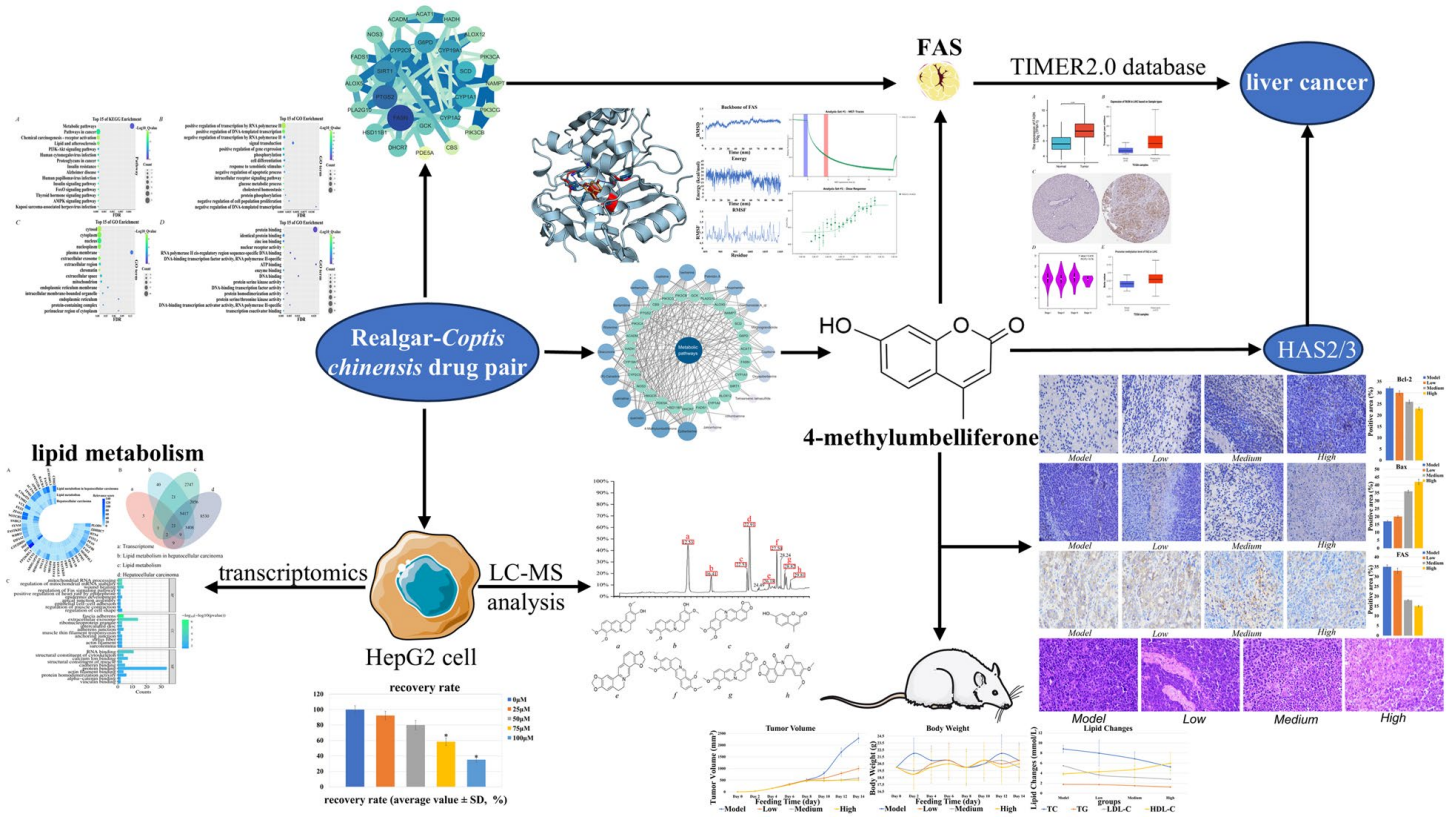

**Supplementary Information:**

The online version contains supplementary material available at 10.1007/s13659-025-00540-9.

## Introduction

Globally, liver malignancies rank sixth in cancer incidence and third in cancer-related mortality [1]. The insidious onset of HCC often results in late-stage diagnoses, precluding curative surgery [2]. Patients thus rely on suboptimal therapies such as chemotherapy, targeted agents, and immunotherapies, which exhibit significant toxicity profiles. Targeting lipid metabolic pathways represents a promising therapeutic strategy, given their central role in HCC progression and metastasis [3, 4]. Lipid metabolism sustains tumorigenesis by regulating cellular growth, energy homeostasis, and signaling cascades. Tumor cells exploit upregulated fatty acid synthesis, catabolism, and biomolecule production to fuel proliferation, invasion, metastatic dissemination, and therapy resistance [5, 6]. Cellular homeostasis is intrinsically linked to lipid metabolic regulation [7]. During tumor progression, nutrient-deprived microenvironments drive cancer cells to depend on lipid metabolism for rapid biomass expansion, survival, and metastatic competence [8, 9].

Modern pharmacological studies have established that Realgar and *Coptis chinensis* induce tumor cell apoptosis [10–13]. traditional Chinese medicine classifies Realgar as a thermogenic and hepatotoxic agent linked to the liver meridian, while *Coptis chinensis* is categorized as a cold-natured, bitter herb acting on the heart and stomach meridians [14, 15]. As a thermogenic agent, Realgar neutralizes hepatotoxins, whereas the cold nature of *Coptis chinensis* exerts antipyretic effects. The synergy between these two agents balances their thermogenic and cold properties, amplifying detoxification efficacy. However, the therapeutic mechanisms of this drug pair in oncology remain incompletely characterized, necessitating systematic exploration.

Studies show that tumor cells often face harsh conditions of low oxygen and nutrition due to connective tissue formation, defective vasculature, and rapid proliferation. Thus, tumor cells adapt metabolically, with lipid metabolism reprogramming being a key part. In normal cells, lipid synthesis is regulated by negative feedback [16]. When intracellular lipid concentration reaches a certain level, synthesis stops to prevent resource waste [17]. In tumor cells, the regulatory mechanism of lipid synthesis fails. Even with normal intracellular lipid levels, lipid metabolism remains highly active, constantly supplying material and energy for rapid proliferation [18]. In normal cells, lipid metabolic pathways maintain basic functions like cell membrane structure, energy storage, and signal transduction [19]. In contrast, tumor cells increase fatty acid utilization through overexpression of fatty acid metabolizing enzymes, supporting their growth, invasion, and metastasis [20]. In normal cells, metabolic enzymes perform traditional functions such as gluconeogenesis and lipid synthesis. However, in tumor cells, metabolic enzymes may acquire new, non-classical functions. These differences in lipid metabolism between HCC and normal cells make inhibiting lipid metabolism a safe and effective strategy for treating HCC [21–23]. In addition, HAS2/3 provides a favorable microenvironment for tumor cells by promoting their growth and metastasis through increased HA synthesis, and its activity is closely related to the extracellular matrix (ECM) in the tumor microenvironment [24, 25]. As an important component of the ECM, HA regulates cell adhesion, migration, and signaling by binding to cell surface receptors such as CD44 [26, 27].

Preliminary transcriptomic analyses demonstrated that the RCCD suppresses HCC through lipid metabolic modulation. Lipid metabolism sustains cellular homeostasis by coordinating fatty acid transport, biosynthesis, storage, and β-oxidation. FAS, a master regulator of lipid metabolism, drives cancer cell proliferation and survival [7]. Hypoxia-inducible factors activate FASN transcription under low oxygen tension, sustaining energy metabolism and membrane integrity in cancer cells [28]. As the sole enzyme catalyzing palmitate synthesis from carbohydrate precursors, FAS generates essential substrates for long-chain and polyunsaturated fatty acid production [29]. Current FAS inhibitor development prioritizes clinical-stage agents like TVB-2640 (targeting solid tumors including glioblastoma, breast, lung, and colorectal carcinomas), whereas preclinical candidates such as cerulenin and C75 remain under investigation [30–33]. FAS inhibitors exhibit broad-spectrum antitumor efficacy across diverse malignancies [34].

Transcriptomics involves the examination of RNA to understand gene expression, focusing on the transcriptional activity and the modulation of the transcriptome at either the cellular or tissue level [35]. The transcriptome includes all RNA molecules transcribed in living cells and is a crucial tool for exploring cellular features and functions. The process of transcription, which involves the use of a DNA template to produce RNA, marks the commencement of gene expression and serves as a pivotal juncture in the regulation of genetic activity [36]. Transcriptomics facilitates the quantification of gene expression across the entire genome within cellular or tissue samples, thereby enabling the assessment of alterations within living organisms under defined experimental settings.

MST is a flexible and potent method for probing the dynamics of biomolecular interactions. It accomplishes this by quantifying the migration of molecules in response to a thermal gradient. This motion, known as thermophoresis, is influenced by changes in hydration shell, charge, or molecular size [37]. MST uses fine capillaries and infrared lasers to create microscopic temperature gradients. The target molecule’s fluorescence changes in response to this gradient, allowing detection of binding events. This technique’s advantage is that it does not require immobilizing the molecule on a surface, allowing measurements in the molecule’s native state. MST is highly sensitive and can detect subtle changes like protein phosphorylation events or small molecule binding [38]. It is suitable for various applications, including analyzing interactions between proteins, DNA, RNA, peptides, small molecules, and ions. Additionally, MST can be used in various buffer conditions and with different sample types, including liposomes, vesicles, and nanoparticles. MST data are plotted against ligand concentration to generate dose–response curves, allowing binding affinities to be inferred. This provides accurate and reliable data on the binding affinity and kinetics of biomolecular interactions [37, 38].

In this study, based on transcriptomic results, the active ingredients of RCCD and their targets were screened by network pharmacology and bioinformatics. The interaction of 4-MU with FAS was verified by MST [39]. The antitumor activity of 4-MU and its effects on lipid metabolic pathways were investigated using the H22 mouse tumor model. The inhibitory effect of 4-MU on HAS2/3, combined with its antitumor activity and effects on lipid metabolic pathways, provides a new potential direction for the treatment of HCC and the development of novel tumor therapeutic strategies.

## Results

### Qualitative analysis

**Fig. 1:**
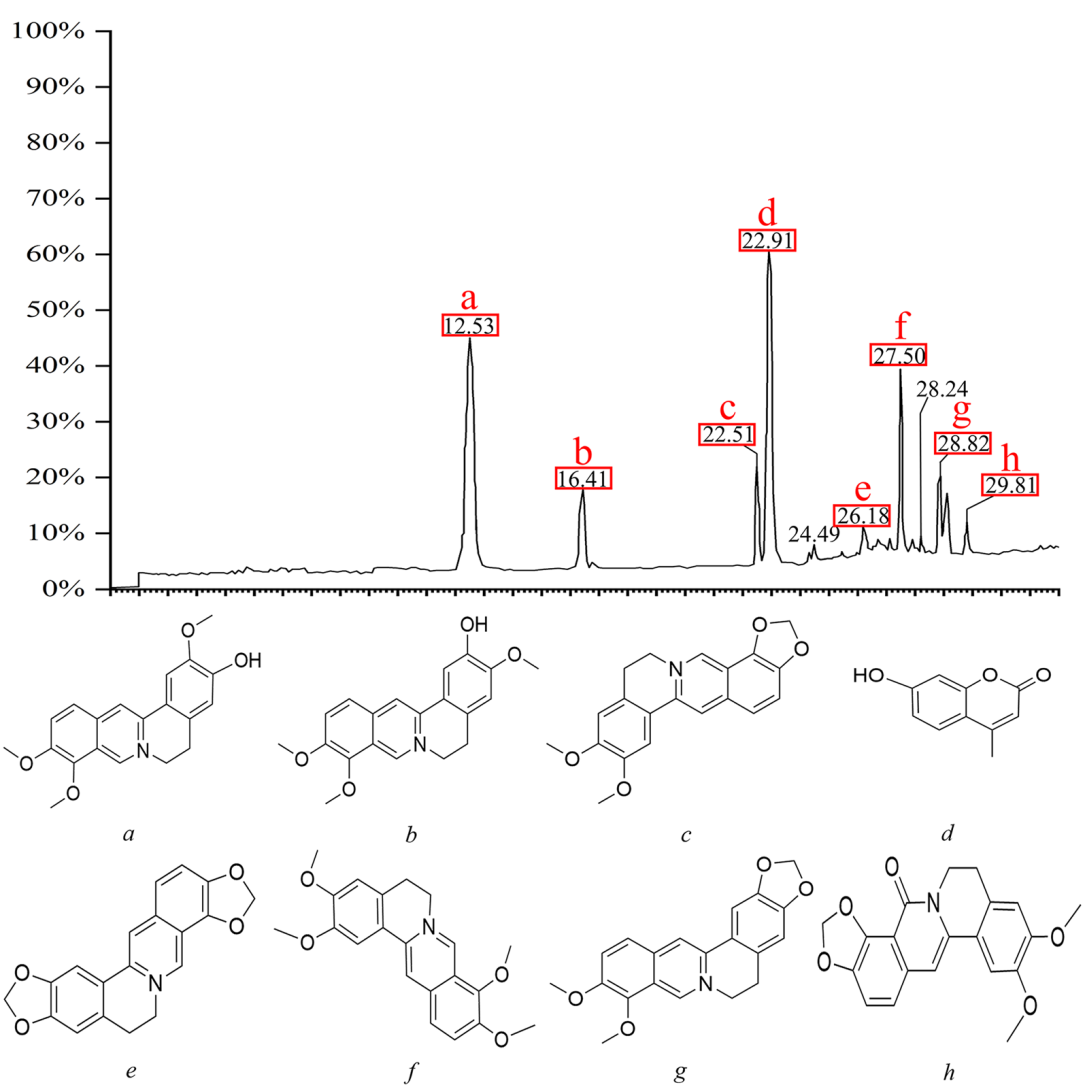
Analysis of active components in RCCD by LC–MS. Liquid chromatogram: The chromatographic peak of the standard of the sample. Peak **a**: Jatrorrhizine; Peak **b**: columbamine; Peak **c**: Epiberberine; Peak **d**: 4-MU; Peak **e**: Coptisine; Peak **f**: palmatine; Peak **g**: Berberine; Peak **h**: Oxyepiberberine

After appropriate chromatographic pretreatment, samples were analyzedby LC–MS. By comparing relative retention time, UV absorption spectra, and the published literature [40, 41], mass spectrometry fragmentation of the chromatographic peaks indicated that Epiberberine, 4-MU, Jatrorrhizine, and other compounds acted as binding ligands (Fig. 1).

### Identification of differentially expressed genes

**Fig. 2:**
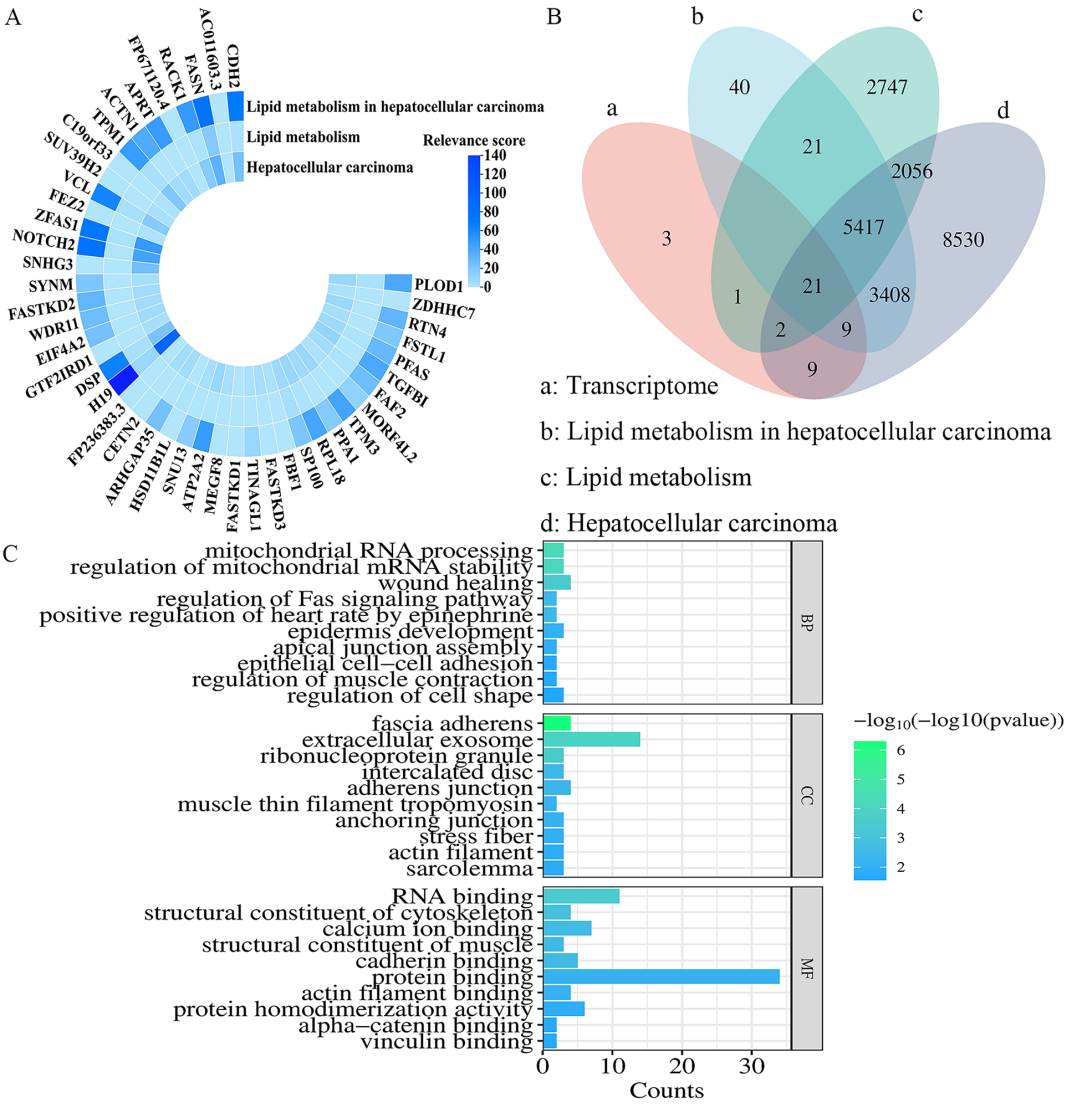
Association of 45 cancer-related differential genes with HCC and lipid metabolism. **A** Correlation between differentially expressed genes and lipid metabolism in HCC from transcriptome data. **B** Differentially expressed genes and HCC, LIHC, and the intersection between lipid metabolism. **C** Top 10 enriched GO terms (BP, CC, MF) for the 42 differentially expressed genes

Differential genes were identified using DEG-seq software, with statistical significance defined as Log2FC(H_O/H) ≥ 2 and P < 0.05. A total of 45 differentially expressed genes highly (Supplementary Table S1). These genes exhibited high correlation with lipid metabolism, and the lipid metabolism pathway was identified as the core pathway for drug interference with tumor cell development (Fig. 2A). which illustrates the correlation between these 45 differentially expressed genes and lipid metabolism in liver cancer (LIHC) [42].

**Fig. 3:**
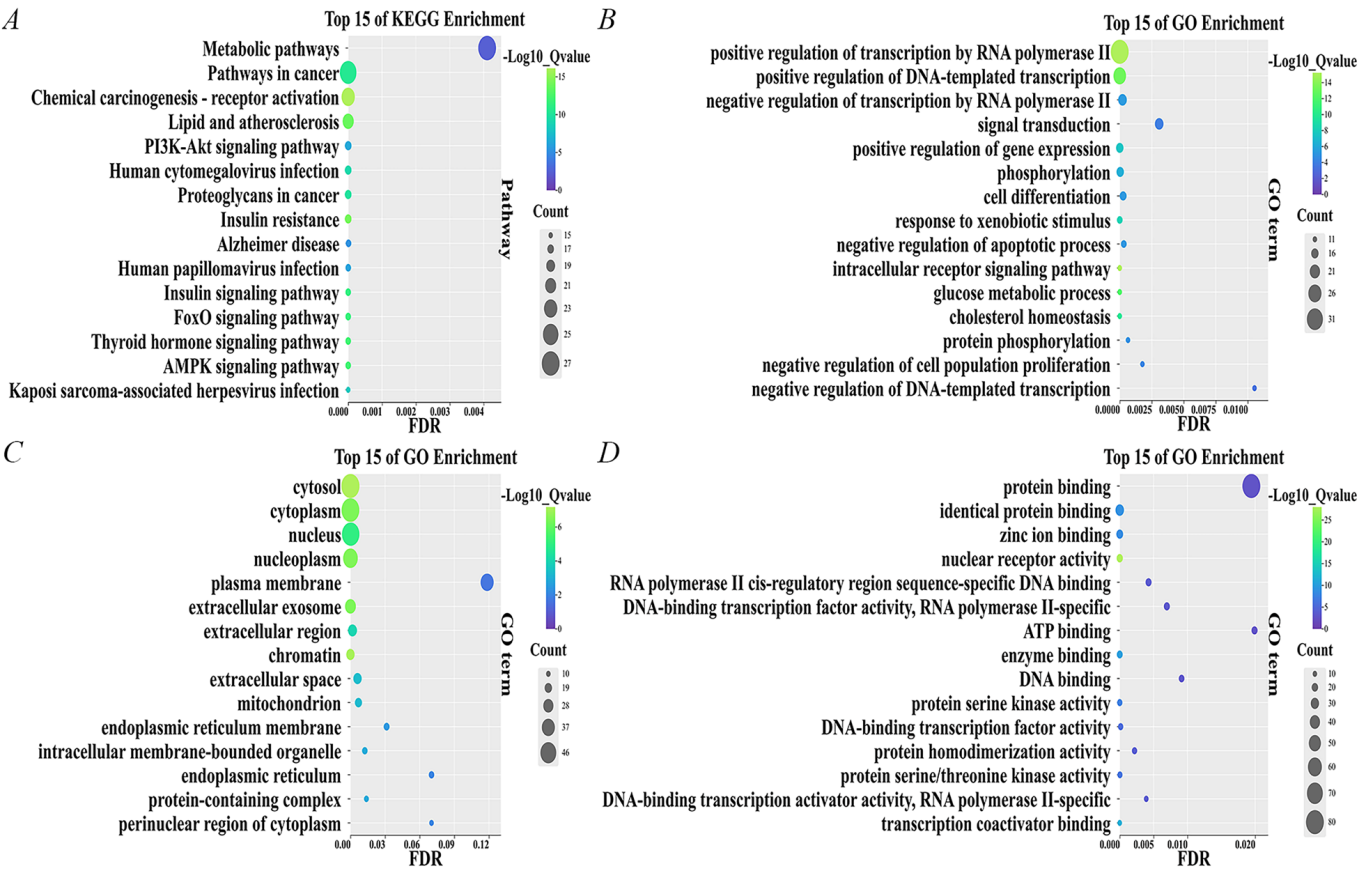
Intersection target enrichment results. **A** Bubble chart of KEGG pathway. **B** Bubble chart of BP. **C** Bubble chart of CC. **D** Bubble chart of MF

Of the 45 differentially expressed genes, 30 were associated with lipid metabolism in HCC, of which the five genes with the highest correlation were H19, FASN, NOTCH2, ZFAS1 and CDH2. 24 were associated with lipid metabolism, of which the five genes with the highest correlation were FASN, H19, SP100, DSP and VCL. 41 were associated with HCC. The five genes with the highest correlation were H19, ZFAS1, NOTCH2, FASN and SNHG3. Notably, FASN showed significant correlation in all three domains (Fig. 2B). After excluding three unrelated genes, the remaining 42 differentially expressed genes were analyzed for Gene Ontology (GO) enrichment (Fig. 3C). The top 10 pathways including biological processes (BP), cellular components (CC), and molecular functions (MF) were illustrated using bar graphs. The most prominent pathway enriched for BP was mitochondrial RNA processing, the most prominent pathway enriched for CC was fascial adhesion, and the most prominent pathway enriched for MF was RNA binding.

### Prediction and analysis of the target genes

Twenty active ingredients were identified based on the results of LC/MS and traditional Chinese medicine databases, as well as relevant literature. Subsequently, the differentially expressed genes were integrated with the target genes of these 20 active compounds, yielding a total of 899 target genes. A total of 611 lipid metabolism target genes were identified and screened from GeneCards. Venn analysis was employed to compare the target genes of the active compounds with those associated with the disease, identifying 101 overlapping target genes (Supplementary Table S2). These intersecting targets were subsequently uploaded to the DAVID database for further analysis. The top 15 pathways, including BP, CC, and MF, were graphically represented using bubble charts (Figs. 3A–D).

The Kyoto Encyclopedia of Genes and Genomes (KEGG) pathway enrichment analysis revealed that all 101 potential target genes were significantly enriched across 158 pathways (Fig. 3A). Notably, 153 of these pathways showed a significant association with the target genes at a P-value of 0.05 or less. The most prominently represented pathways were Metabolic pathways (26.73%), Pathways in cancer (25.74%), and Chemical carcinogenesis—receptor activation (22.77%).

GO enrichment analysis revealed that all 101 genes (100%) were associated with BP, CC, and MF. Specifically, BP enrichment was mainly associated with: positive regulation of transcription by RNA polymerase II (33.66%), positive regulation of DNA-induced transcription (24.75%), negative regulation of transcription by RNA polymerase II (17.82%), and signal transduction (17.82%) (Fig. 3B). CC enrichment was mainly associated with: cell membrane (52.48%), cytoplasm (51.49%), nucleus (50.49%), and nucleolus (41.58%) (Fig. 3C). MF enrichment was mainly associated with: protein binding (79.21%), isoform protein binding (30.69%), zinc ion binding (21.78%), and nuclear receptor activity (18.81%) (Fig. 3D). KEGG and GO enrichment analysis of overlapping targets may provide valuable information for understanding the antitumor and anti-HCC properties of RCCD.

The bioconcentration data indicate that the mechanism of action of RCCD involves the binding to specific proteins, thereby modulating their functions, regulating associated metabolic pathways, and influencing DNA expression to ultimately affect protein synthesis.

### Network construction

**Fig. 4:**
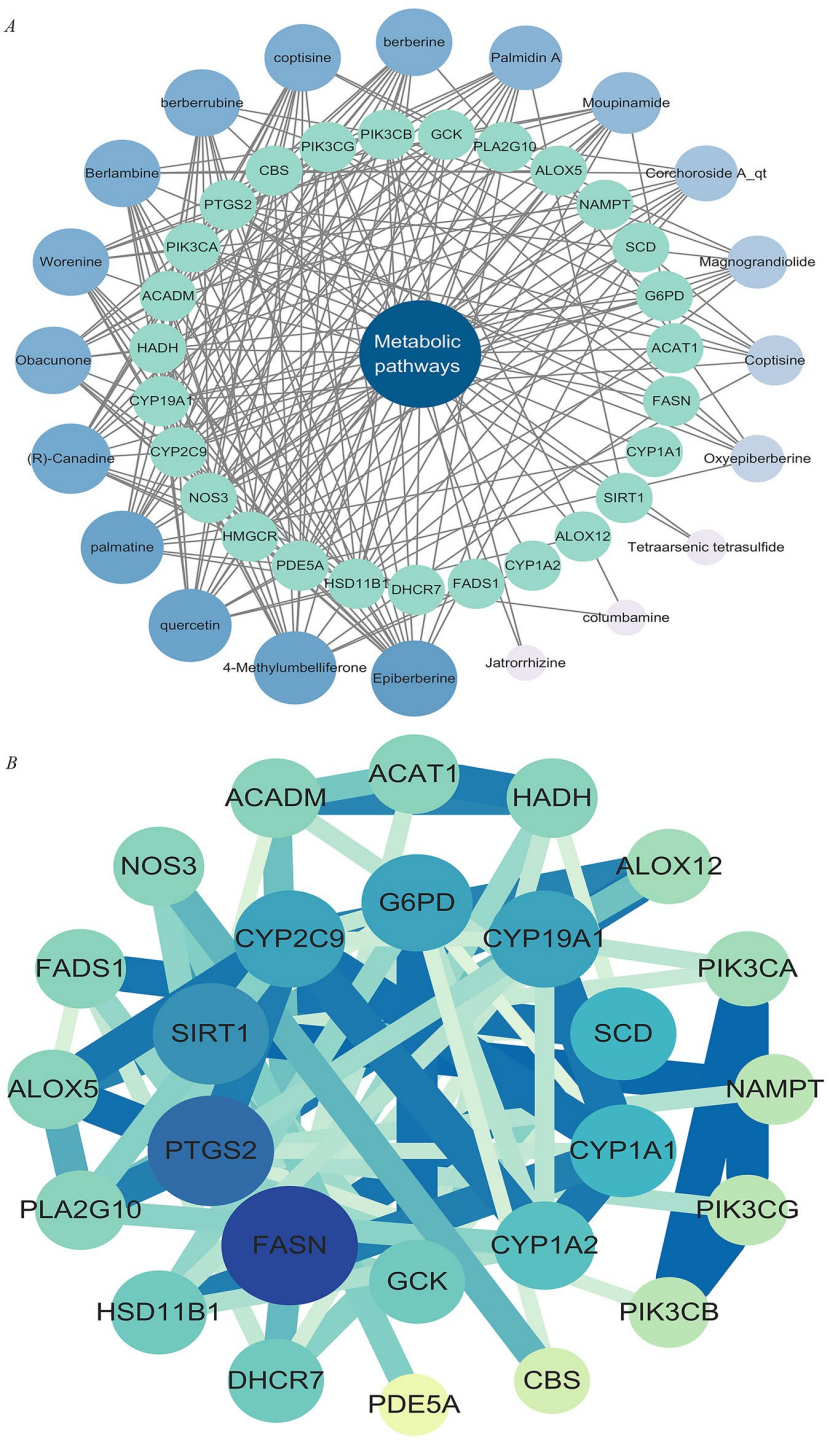
network analysis. **A** “core pathway-compound-target” network. **B** “PPI” network

Targets enriched in the top KEGG pathway, Metabolic pathways, and 20 active compounds were imported into Cytoscape 3.10.1 to construct a “core pathway-compound-target” network for core signaling pathways (Fig. 4A). The network diagram illustrated the combined impact of various compounds on multiple targets within key signaling pathways when the RCCD was used to modulate lipid metabolism. By examining the degree values within this network, it was possible to pinpoint high-degree nodes that could represent potential active compounds (Table 1). This approach suggests that the compounds associated with the highest degree values may have a significant influence on the network, indicating that they could be crucial in the therapeutic effects of the drug pair on lipid metabolism. These high-degree nodes are likely interacting with multiple targets, which may contribute to the overall synergistic effect observed in the treatment. It can be considered that when the RCCD exerts anti-cancer effects through lipid metabolism, high-degree compounds play a crucial role in the core pathways.

**Table 1:** The top 8 active compounds and potential targets

Compound	Degree	Gene	Degree
Epiberberine	16	FASN	15
4-MU	15	PTGS2	14
Quercetin	15	SIRT1	12
Palmatine	15	CYP19A1	10
(R)-Canadine	14	CYP2C9	9
Worenine	14	G6PD	9

After the identification and concentration of key elements within the central pathways, these elements were subsequently uploaded to the STRING platform for further analysis. Subsequently, a Protein–Protein Interaction (PPI) network was meticulously constructed using the software Cytoscape 3.8.2 (Fig. 4B). This PPI network provides a visual representation of the interactions between proteins, which is instrumental in understanding the complex BP at play. The network can help identify key proteins that may be central to the pathways targeted by the RCCD, offering further insights into their potential synergistic effects on lipid metabolism. Core targets were identified by analyzing the degree values of the network diagram (Table 1). These targets play crucial roles in the core signaling pathways.

Within the PPI network, FASN (degree = 15) emerged as the highest-connectivity core target. This pivotal enzyme orchestrates de novo lipogenesis in HCC, fueling pathogenic palmitate accumulation via the HBx-SREBP1c transcriptional axis to sustain tumor bioenergetics and membrane dynamics [43]. PTGS2 (degree = 14) amplifies inflammatory cascades by converting FASN-derived arachidonic acid into protumorigenic prostaglandin E_2_, establishing a self-perpetuating lipid-inflammation feedforward loop that drives IL-6/STAT3-mediated malignant transformation in non-alcoholic steatohepatitis [44, 45]. SIRT1 (degree = 12) functions as a context-dependent metabolic rheostat, exhibiting stage-specific duality: during early hepatocarcinogenesis it activates AMPK to suppress SREBP1c/FASN-driven lipogenesis, whereas in advanced HCC it stabilizes HIF-1α through deacetylation to potentiate glycolytic flux and lipid droplet storage [46, 47].

The “active ingredient-target” network was utilized to identify Epiberberine, 4-MU, quercetin, and palmatine as the core active ingredients with the highest degree of activity (Table 1).

### FAS protein expression in HCC

**Fig. 5:**
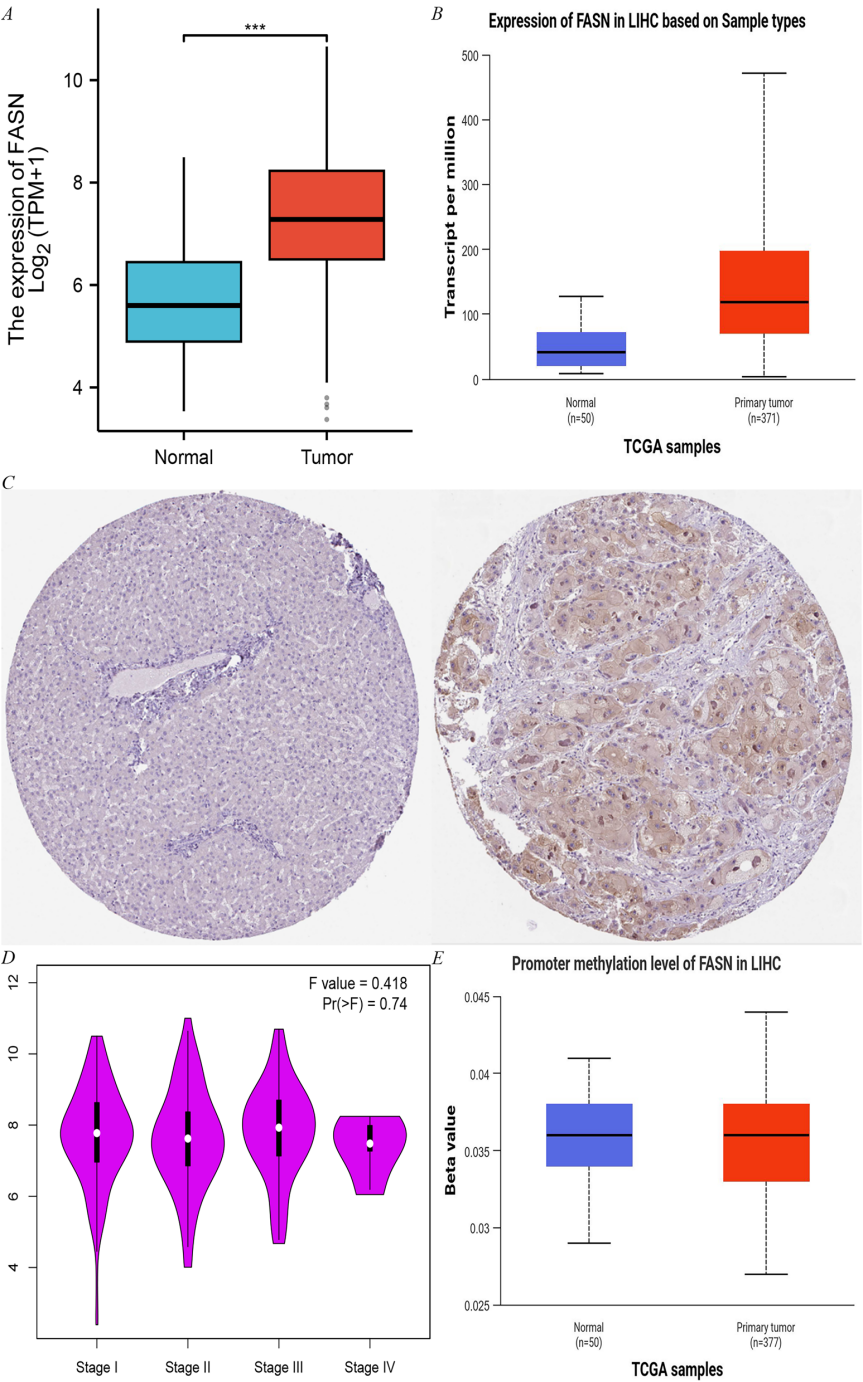
The expression of FASN in LIHC-Tumor. **A** FASN expression in LIHC-Tumor from TIMER2.0. **B** The protein levels of FAS in LIHC were analyzed using CPTAC. **C** Comparison of FASN expression differences between LIHC tumor tissues and normal tissues from TCGA database. **D** FASN expression levels and the pathological stages were analyzed using GEPIA2.0. **E** Correlation between methylation level and expression of FASN promoter

The results of biosignature analysis demonstrate that FASN mRNA is significantly overexpressed in HCC tissues (Fig. 5A), and its protein level is also significantly upregulated (Fig. 5B). FAS protein expression was elevated approximately 2.5-fold in tumor tissues compared with normal liver tissues (p < 0.001) (Fig. 5C). Further analysis revealed that FASN expression was positively correlated with the pathological stage of HCC (F = 0.418, p = 0.74) (Fig. 5D). Methylation analysis revealed that the methylation level of the FASN promoter region was negatively correlated with gene expression (r = − 0.21, p < 0.05), suggesting that DNA methylation may regulate the aberrant expression of FASN (Fig. 5E).

### Binding of 4-MU to FAS and its effect on HepG2 cells

**Fig. 6:**
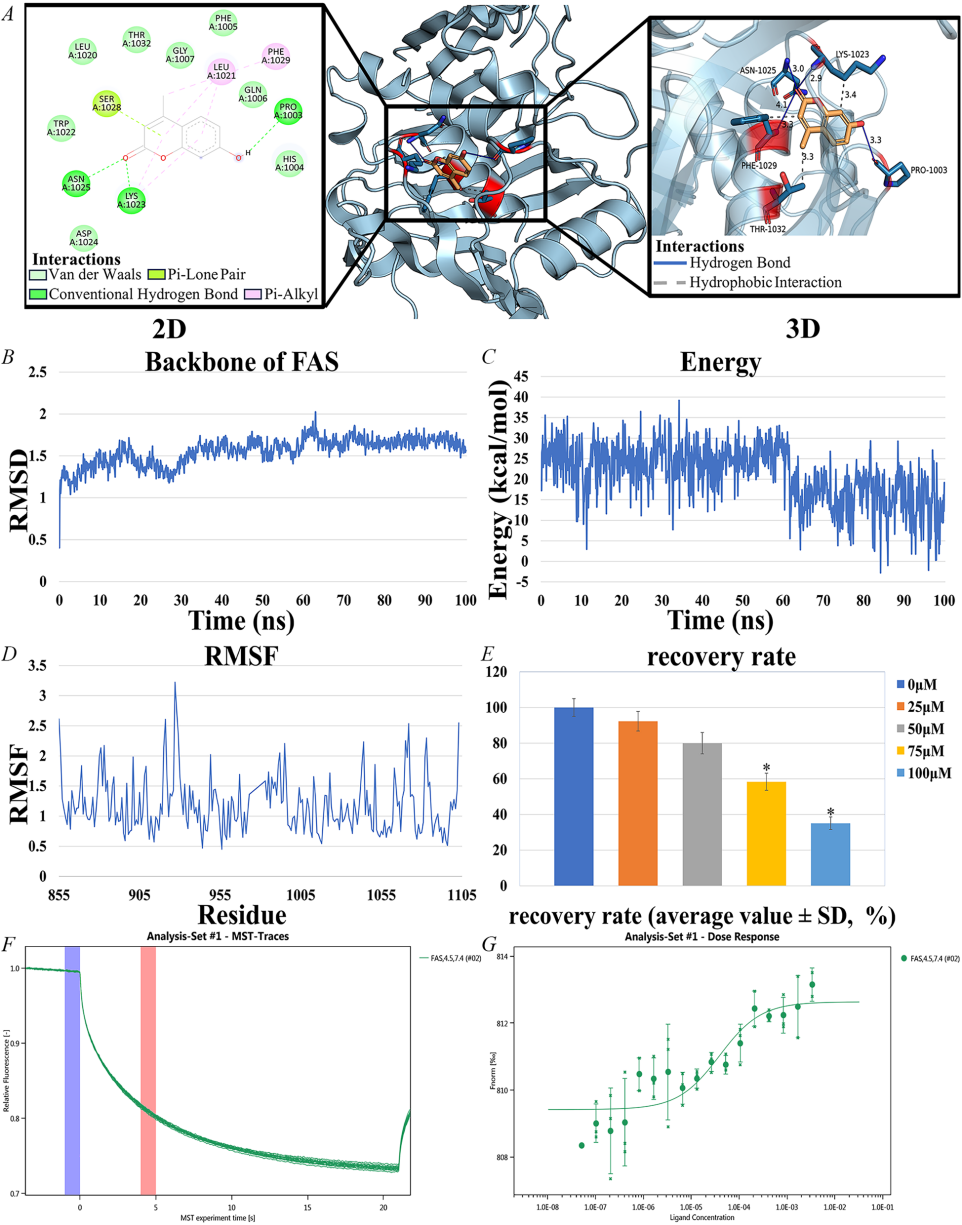
interaction of 4-MU with FAS and Results of HepG2 cell experiments. **A** Docking results of 4-MU with FAS. **B** RMSD of 4-MU and FAS complexes. **C** Energy of 4-MU and FAS complexes. **D** RMSF of 4-MU and FAS complexes. **E** Results of HepG2 cell experiments, IC50 = 85.4 μM, 95% confidence interval of 80.2–90.6 μM. **F**, **G** Molecular interactions of 4-MU by NT. 115 analysis. FAS (0.338 μM), 4-MU ranged from 3.38 mM to 5.15E-05 mM, Kd = 38.755 nM

Molecular docking was performed using AutoDock, and the results were evaluated based on the free energy of binding. The free energy of binding, typically expressed in kcal/mol, represents the energy released when a ligand binds to a receptor. Binding free energies below − 1.2 kcal/mol are generally considered indicative of effective binding. The top four active ingredients and FAS were selected for docking. among all the active ingredients bound to FAS, 4-MU exhibited the lowest binding free energy (− 6.91 kcal/mol), indicating a more stable binding interaction (Table 2). This suggests that 4-MU is the most likely core active ingredient to bind to and function with FAS (Fig. 6A).

**Table 2:** Dock binding free energies (△Gb) and bonds of the docked inhibitors against FAS

PDB code	Inhibitors	Gb (kcal/mol)	Bonds between groups of compounds and amino acids of FAS		
			Groups of comp	Amino acid	Bonds name
8g7x	4-MU	− 6.91	O	ASN1025	H-bond
			O	LYS1023	H-bond
			O	PRO1003	H-bond
			O	PHE1029	H-bond
			Six-member	SER1028	Pi-Pi
			Six-member	PHE1029	Hydrophobic
			Ring Benzene	LYS1023	Hydrophobic
			C	THR1023	Hydrophobic
	Epiberberine	− 6.51	H	ASP962	C-H-bond
			O	ARG942	C-H-bond
			H	LEU938	C-H-bond
			H	ALA944	C-H-bond
	Palmatine	− 6.12	H	THR1087	C-H-bond
			H	LEU1097	C-H-bond
			O	SER1037	H-bond
			O	ARG1082	H-bond
	Quercetin	− 5.84	H	ALA943	H-bond
			H	ASP962	H-bond

Root mean square deviation (RMSD) analysis quantified structural deviations from the initial conformation during simulation. The trajectory exhibited minor fluctuations but maintained overall stability with minimal backbone conformational changes (Fig. 6B). Energy fluctuation analysis revealed initial high energy (19.054 kcal/mol) that gradually decreased and stabilized, indicating system convergence to a low-energy equilibrium state (Fig. 6C). Root mean square fluctuation (RMSF) analysis assessed residue-specific flexibility, showing significant variation across protein regions that highlighted differential mobility during simulation (Fig. 6D). Overall, the binding of 4-MU to FAS was stable, and the complex maintained a consistent conformation and lower energy state throughout the simulation. The ADME properties of 4-MU (Table 3), indicate favorable intestinal absorption, high water solubility, and good drug-forming properties.

**Table 3:** ADME properties of 4-MU

Lipophilicity	Water solubility	Pharmacokinetics	Druglikeness
Consensus Log *P*_*o/w*_ = 1.81	log S (ESOL) = − 2.70	GI absorption: High	Lipinski: Yes; 0 violation
	Class (ESOL): Soluble	BBB permeant: Yes	Bioavailability Score = 0.55

A dose-dependent effect of 4-MU on HepG2 cell viability was observed. The IC50 value of 4-MU for HepG2 cells was 85.4 μM, with a 95% confidence interval of 80.2–90.6 μM. This indicates that 4-MU can produce significant inhibitory effects on HepG2 cells at moderate concentrations (Fig. 6E).

The interaction between 4-MU and FAS was evaluated using MST, a technique that quantifies molecular affinity. The dissociation constant (Kd) was determined by assessing the normalized fluorescence intensity variance between the ligand-receptor binding complex and the unbound ligand, which allowed for the subsequent calculation of the binding fraction.

In the process, a range of concentrations of the active compound was introduced to the target protein, and the MST instrument monitored the fluorescence signal change as the molecules experienced thermal motion within a temperature gradient. This change in fluorescence signal was amplified by a factor of 1000 and expressed as relative fluorescence units. Following that, the Kd was determined from the standardized variation in fluorescence signal, comparing the states where the ligand was bound versus unbound to the protein. The findings indicated a robust binding affinity of 4-MU for the target protein FAS, as detected by MST (Figs. 6F and G).

### Results of in vivo anti-tumor assay

**Fig. 7:**
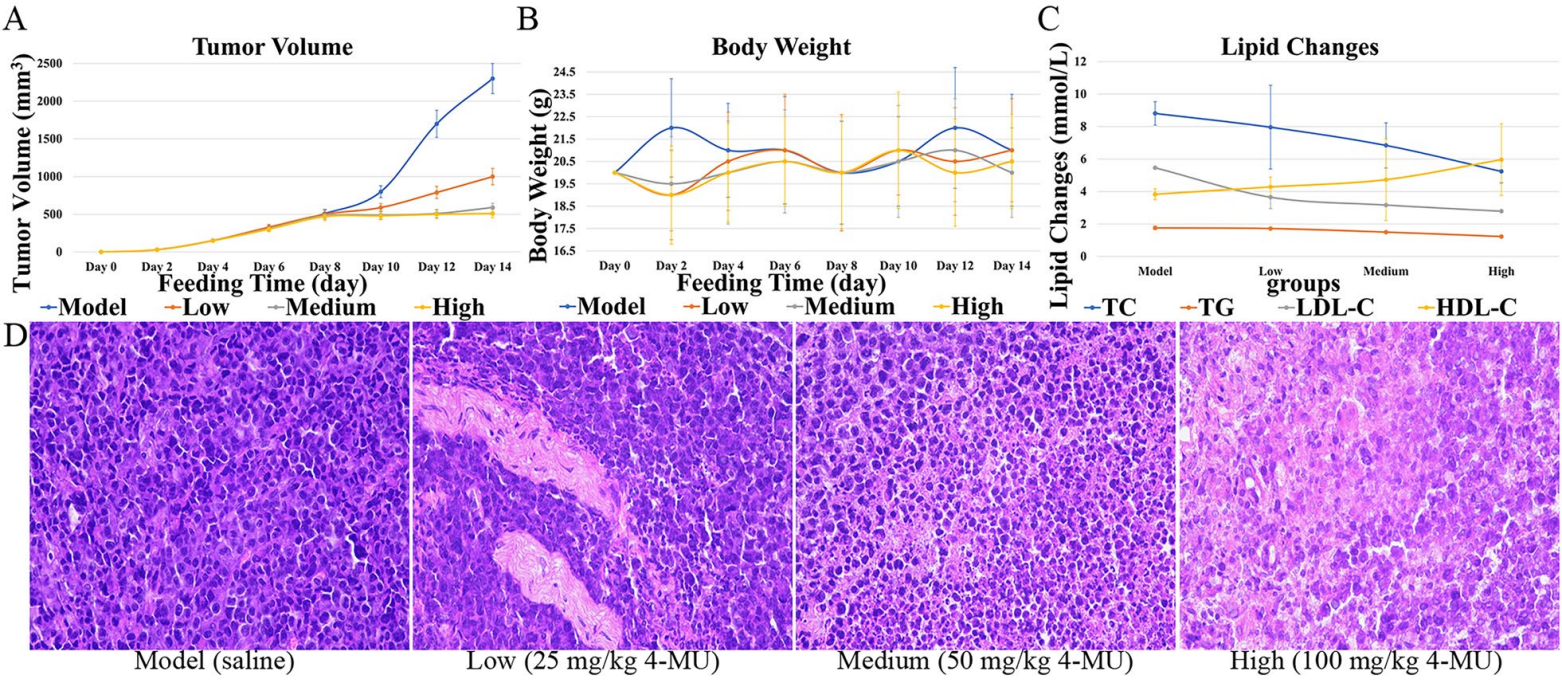
Effects of 4-MU on mouse xenograft models and tumor tissues. **A** Tumor volume. **B** Body weight. **C** Lipid Changes. **D** HE image of tumor tissue (original magnification 400 ×)

**Fig. 8:**
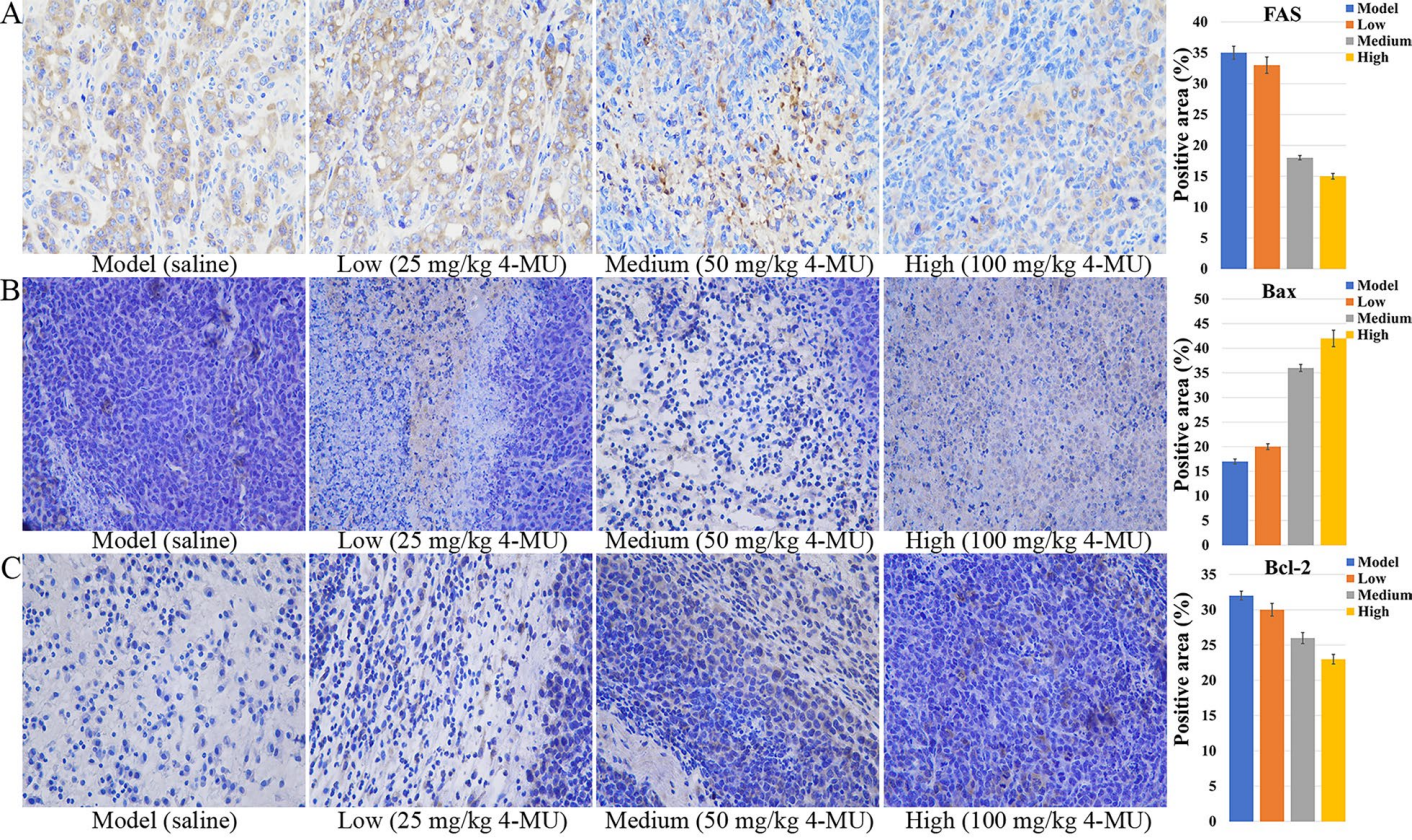
Effect of 4-MU on protein expression in tumor tissues of a mouse xenograft model. **A** ICH image of FAS (original magnification 400 ×). **B** ICH image of Bax (original magnification 400 ×). **C** ICH image of Bcl-2 (original magnification 400 ×)

The antitumor efficacy of 4-MU was evaluated in BALB/c mice bearing H22 tumor xenografts. After tumor implantation, mice were weighed every two days, and tumor size was measured using digital calipers to calculate tumor volume (Figs. 7A and 7B). After 14 days, mice were euthanized, and blood was collected for lipid content analysis (Fig. 7C). Tumor tissues were excised from the mice for histopathological sectioning (Fig. 7D). Immunohistochemical analyses of tumor tissues were conducted (Figs. 8A, B, and C).

The dose-dependent effects of 4-MU on tumor progression and body weight changes in tumor-bearing mice were assessed. By day 10, tumor growth in the control group showed a marked acceleration (P < 0.05 compared to baseline). Conversely, 4-MU treatment resulted in dose-dependent tumor suppression, with significant growth inhibition observed at all tested concentrations (P < 0.01 vs. control) (Figs. 7A and B). The dose-responsive alterations in serum lipid profiles following 4-MU administration were observed. Dose-dependent reductions were observed in TC, TG, and LDL-C levels, whereas HDL-C concentrations showed a progressive increase across treatment groups. These lipid-modulating effects were mediated through 4-MU's inhibition of FAS activity, which subsequently attenuated fatty acid biosynthesis and regulated cholesterol homeostasis (Fig. 7C).

The effects of 4-MU on mouse tumor cells were observed. In tumor tissues, cell density decreased with increasing drug dose; cell arrangement became more disorganized; and tissue necrosis or abnormal cell proliferation was observed, with nuclei stained dark purple and cytoplasm stained pink (Fig. 7D).

4-MU treatment induced a concentration-dependent suppression of FAS protein expression, as visualized by diminished yellow–brown immunohistochemical staining intensity (Fig. 8A). Consistent with its role in apoptosis modulation, 4-MU significantly altered critical regulators of cell death in H22 tumor-bearing mice: Bax expression progressively intensified with escalating drug concentrations (Fig. 8B), whereas Bcl-2 expression exhibited reciprocal attenuation (Fig. 8C). This concerted dysregulation culminated in a dose-responsive decline of the Bcl-2/Bax ratio, functionally demonstrating enhanced tumor cell apoptosis through mitochondrial pathway activation.

## Discussion

Modern studies have found that Realgar and *Coptis chinensis* are cytotoxic, inducing tumor cell death and inhibiting tumor growth and survival [11, 48–50]. Active lipid metabolism in HCC cells supports their rapid survival and proliferation in the harsh microenvironment, playing a crucial role in tumor development [16, 51]. Based on LC/MS and transcriptomics data, the “Core Pathway-Compound-Target” network and the PPI network were established using a network pharmacology approach. This study investigated the active ingredients, potential target genes, and related signaling pathways of RCCD in anti-HCC, focusing on lipid metabolism to elucidate its possible mechanism of action. Then, the network pharmacology results were validated through MST experiments, as well as in vitro and in vivo studies.

Through data screening and analysis, 20 active compounds, core signaling pathways, and 27 potential target genes related to the core pathways were identified. These may represent the mechanisms by which the RCCD intervenes in lipid metabolism. GO functional and KEGG pathway enrichment analyses identified 101 overlapping target genes that are implicated in various cellular compartments, including the cytosol, cytoplasm, and nucleus. Molecularly, these overlapping targets were engaged in processes such as protein binding, homotypic protein binding, and nuclear receptor activity. They pertained to the facilitation of transcription by RNA polymerase II, the enhancement of DNA-templated transcription, and the suppression of transcription by RNA polymerase II. The results suggest that 4-MU may inhibit tumor cell proliferation and promote tumor cell death by binding to proteins in the cytoplasm and affecting the expression of lipid metabolism-related genes. 4-MU may exert its anti-leukemia effects by inhibiting key targets of lipid metabolism. Analysis of the PPI network and the “core pathway-compound-target” network, as well as molecular docking, identified 4-MU as a potential active compound and FASN as a potential target gene related to lipid metabolism.

FAS, a pivotal enzyme in the de novo synthesis of fatty acids, exerts a critical rate-limiting influence on the intracellular fatty acid synthesis pathway. It is essential for maintaining the energy metabolism and structural homeostasis of cancer cell membranes [29]. FAS is the only enzyme capable of converting carbohydrate metabolites into palmitate, a key component of long-chain and polyunsaturated fatty acids. Fatty acids are vital components of cell membranes, and FAS maintains their integrity and function by synthesizing fatty acids. In various malignancies, including breast, prostate, and lung cancer, FAS expression is notably increased. Given that tumor cells demand substantial fatty acid supplies to fuel their accelerated growth and proliferation, FAS inhibition emerges as a promising therapeutic strategy against cancer [52, 53].

The Bcl-2 family can be categorized into two subgroups: proteins that oppose apoptosis and those that promote it. Bcl-2 and Bax are considered the key representatives of the anti-apoptotic and pro-apoptotic factions, respectively. In most tumors, Bcl-2 expression is elevated, while Bax expression is reduced. Reducing Bcl-2 levels or increasing Bax levels can stimulate the process of apoptosis in a variety of cancer cells. The balance between Bax and Bcl-2 expression is significantly associated with the growth and advancement of tumors [54].

MST assays reveal a strong binding interaction between 4-MU and FAS. Molecular docking and MD simulations indicate that the 4-MU-FAS complex exhibits high stability. In vivo studies assessing the antitumor efficacy of 4-MU revealed that changes in mouse body weight indicated no significant adverse effects on overall health. The dose-dependent reduction of total blood cholesterol in mice demonstrated the inhibitory effect of 4-MU on lipid metabolism pathways. Since the synthesis of triglycerides and LDL cholesterol depends on fatty acids, as the dose of 4-MU increases, triglycerides and LDL cholesterol decrease, while HDL cholesterol increases. Therefore, as the dose of 4-MU increased, triglycerides and LDL cholesterol decreased, while HDL cholesterol increased, likely due to rebalancing of cholesterol metabolism from FAS inhibition. Tumor suppression rates and histological findings indicate that 4-MU inhibits tumor proliferation with mild efficacy at low doses and significant efficacy at high doses.In vivo studies have shown that moderate to high concentrations of 4-MU can inhibit tumor expansion, suppress lipid metabolism activity, improve the health status of tumor-bearing mice, and alter the cellular appearance and structure of tumors. By binding to FAS protein, 4-MU severely disrupts lipid metabolism. This action causes an energy metabolism crisis and imbalances cell membrane structure in tumor cells. Additionally, the accumulation of acetyl-coenzyme A, a precursor for fatty acid synthesis, activates acetylation modification, which induces tumor cells to enter the pro-apoptotic signaling pathway, ultimately leading to apoptosis. In conjunction with network pharmacology results, 4-MU was found to inhibit FAS activity. This inhibition disrupts the cell membrane and subsequent fatty acid-based energy supply of tumor tissues, thereby inhibiting tumor growth and inducing apoptosis [23, 55, 56].

Notably, 4-MU was reported as a hyaluronic acid synthase inhibitor in previous studies, which significantly reduced the synthesis of hyaluronic acid (HA) in the ECM by inhibiting the expression of HAS2/3, thereby interfering with HA-dependent tumor microenvironment construction and fibrosis progression [57–59]. Combined with its inhibitory effect on FAS, this multi-target property allows 4-MU to exhibit synergistic anti-tumor and anti-fibrosis effects independent of dietary regulation. The dual targeting of FAS and HAS2/3 by 4-MU elicits synergistic anti-tumor effects in HCC. This profound synergy originates from reciprocal reinforcement within a metabolic-ECM signaling loop: FAS-derived palmitoyl-CoA activates SREBP2-mediated HAS2 transcription while generating UDP-GlcNAc—the essential HA precursor. Reciprocally, HA-CD44 complexes facilitate lipid raft assembly that potentiates EGFR/IGF1R-PI3K/AKT signaling, driving SREBP1c phosphorylation to amplify FASN expression and lipogenesis [29, 52, 53, 60, 61]. 4-MU dismantles this vicious cycle through dual mechanisms: depleting palmitoyl-CoA pools to disrupt SREBP2-HAS2 axis activation, and directly suppressing HA synthesis. This coordinated action collapses CD44-EGFR signalosomes and abrogates AKT-dependent SREBP1c maturation, inducing metabolic catastrophe through simultaneous lipid substrate deprivation and survival signal ablation. The therapeutic intervention transcends additive effects by eroding HCC's adaptive infrastructure, achieving tumor regression through coordinated disruption of oncogenic metabolism and stromal protection. This "dual-hit" strategy concurrently inhibits malignant proliferation and stromal support, with preclinical evidence confirming enhanced therapeutic efficacy when targeting both metabolic and microenvironmental pathways in solid tumors [62, 63].

However, 4-MU’s systemic effects may be accompanied by a risk of immunosuppression, and its poor water solubility limits clinical translation. In contrast, TVB-2640 is a highly selective direct inhibitor of FAS. TVB-2640 has been proved to significantly reduce liver fat content (ΔMRI-PDFF≈30%) and inhibit tumor growth in non-alcoholic steatohepatitis and solid tumors in phase II clinical trials, but its mechanism of action is simple and it lacks the ability to regulate ECM remodeling [33, 64, 65]. This mechanistic comparison suggests 4-MU's multitargeted profile offers superior therapeutic potential for complex pathologies like fibrotic tumor microenvironments, whereas TVB-2640's selectivity suits metabolic-disorder-driven malignancies.

## Conclusion

In summary, our study demonstrated that 4-MU, the active ingredient in RCCD, binds to the lipid metabolism-related target FAS, inhibiting fatty acid synthesis and disrupting the balance of cellular membrane structure and energy supply. This inhibition reduced tumor growth in a mouse xenograft model without significantly affecting the overall health of the mice. Moreover, due to its inhibitory effect on HAS2/3, 4-MU may be more advantageous in treating tumor-fibrosis co-morbidities than FAS inhibitors alone, such as TVB-2640. This finding provides a theoretical basis for developing anticancer drugs that target lipid metabolism.

## Materials and methods

### Chemical and reagents

4-MU (CAS 90-33-5), was sourced from Shanghai Yuanye Bio-Technology Co., Ltd. (China), and was provided with a purity of no less than 98%. The FAS (ab131929), Bcl-2 (ab182858) and Bax (ab81083) was procured from Abcam, located in Cambridge, UK.

### Cell culture and binding component screening

HepG2 cells, sourced from the Cell Bank of the Chinese Academy of Sciences, were maintained in a cell incubator under a 5% CO_2_ atmosphere at a temperature of 37 °C, utilizing a specialized HepG2 cell culture medium provided by Procell Life Science & Technology Co., Ltd. Cells that had grown to confluence were transferred into 6-well plates. The control group was maintained in the standard culture medium, while the experimental group was treated with a medium containing a mixture of Realgar and *Coptis chinensis*, supplied by the Hubei Key Laboratory of Chinese Medicine Resources and Chemistry. A portion of the incubated and non-incubated cells was reserved for transcriptome sequencing analysis (Sect. 5.4).

Total proteins were ex-tracted from cells using RIPA buffer with protease/phosphatase in-hibitors (Beyotime Biotechnology, China). 0.11 g/mL methanol extract of the mixture (8.54 g of the mixture were obtained from the Key Laboratory for Traditional Chinese Medicine Resources and Chemistry of Hubei Province) was dissolved to 11 mg/mL by 0.5 M potassium Sephosphate buffer (pH 7); 12 μL of total protein, 60 μL of methanol Dextract and 168 μL of 0.5 M potassium phosphate buffer (pH 7) were stmixed and incubated at 37 °C for 60 min. After incubation, the mixture Qwass filtered for 30 min using an ultrafiltration centrifugal tube (Millipore) at 6350 g and 4 °C, and then washed twice with potassium phosphate buffer to remove unbound compounds. The protein complexwhich was retained on the membrane of the ultrafiltration device wasthen transferred to a new centrifuge tube.

### LC–MS analysis

The 0.22 pm filtration membrane was used to produce a stocksample solution which was analyzed by HPLC-DAD-ESI-MS. Chromatography was performed with a Hanbon LichrospherTM highperformance liquid chromatography C18 column (4.6–250 mm, 5 pm) at 25 C. The mobile phase consisted of solvent A (acetonitrile) and solvent B (0.03 mol/L ammonium bicarbonate aqueous solution). The gradient elution program was set as follows: 0–12 min, 10–25% A; 12–20 min, 25–27% A; 20–32 min, 27–45% A; 32–35 min, 45% A maintained. It's 10 μL permilliliter per minute. The detection wavelength is 190–690 nm. TheSettings of mass spectrometry are as follows; turbine spray temperature:550 °C, source voltage: 5.5 kv for positive ion mode and − 4.5 kv fornegative ion mode. M/Z varies from 50 to 1600. Compounds wereidentified by their precise mass, mass/mass ion fragment patterns andretention time in liquid chromatography.

### Transcriptome sequencing analysis

Total RNA was extracted from both groups following the protocol of the TruSeq™ RNA Sample Preparation Kit from San Diego, CA. RNA samples of high integrity (5 μg, with an OD260/280 ratio of 1.8–2.2) were selected for the construction of sequencing libraries. mRNA with polyA tails was captured from eukaryotic mRNA using magnetic beads coated with Oligo (dT). The captured mRNA was then randomly fragmented into approximately 300 bp segments by the addition of a fragmentation buffer. Subsequently, single-stranded cDNA was synthesized using reverse transcriptase, resulting in a stable double-stranded structure.

The adapter-ligated samples underwent purification and size selection, followed by PCR amplification to selectively enrich the desired products, resulting in the formation of the final library. Subsequently, these libraries were prepared for sequencing using the Illumina HiSeq Xten/Nova-Seq 6000 platform.

Rotating Scanning Electron Microscopy was utilized to perform a quantitative assessment of the transcript and gene expression levels across various samples, highlighting disparities in expression profiles. A differential gene expression analysis was then carried out to pinpoint and filter out genes that exhibited significant changes in expression levels. Subsequently, an in-depth investigation was conducted to explore the specific roles and functions of these differentially expressed genes [66].

### Cross-target gene prediction

The bioactive components of Realgar and *Coptis chinensis* were identified via the TCMSP database (https://old.tcmsp-e.com/tcmsp.php) and combined with the results of LC–MS analysis. The targets of these bioactive ingredients were then identified using PubChem (https://pubchem.ncbi.nlm.nih.gov/), SwissTargetPrediction (http://www.swisstargetprediction.ch/), and the PharmMapper Server (http://PharmMapper.rcas.sinica.edu.tw/steppp) the targets of these bioactive ingredients were then identified. Proteins related to lipid metabolism were pinpointed with the help of GeneCards (https://www.genecards.org/). Subsequently, the intersecting targets were subjected to the DAVID database (https://david.ncifcrf.gov/) to perform GO functional categorization and KEGG pathway enrichment analysis.

### Network construction

Based on KEGG enrichment results, pathways were ranked by enrichment values, core pathways were screened, and related targets were imported into STRING for analysis. A PPI network was constructed to identify core targets. Active compounds and core pathway enrichment targets were uploaded to Cytoscape 3.10.1 to construct a “core pathway-compound-target” network for core signaling pathways [67]. Active compounds and enriched targets were input as nodes, with edges representing connections between them. By analyzing the network diagram, highly targeted compounds were identified.

### Integrated analysis of FAS expression and methylation in HCC using public databases

The analysis of the differential expression of FASN mRNA in LIHC tumors and normal tissues in the TCGA cohort was performed using TIMER2.0 [68]. The expression of FAS protein in HCC was verified based on CPTAC database [69]. The gene expression data of HCC tumor tissues (n = 371) and normal liver tissues (n = 50) were obtained by TCGA database, and the independent sample t-test was used to compare the differences of FAS between the two groups. The correlation between FASN expression and the pathological stage of HCC (stage I-IV) was analyzed with the help of GEPIA2.0 platform, and one-way analysis of variance (ANOVA) was used to assess the differences between groups. Methylation data of HCC samples were extracted using the UCSC Xena database to analyze the correlation between the methylation level (β-value) of FASN promoter region and gene expression, and Pearson correlation coefficients were calculated.

### Binding of 4-MU to FAS and its effect on HepG2 cells

To explore the binding affinity between specific bioactive substances and their prospective targets, a computational docking process was undertaken employing AutoDockTools (version 1.5.7). The 3D chemical structures for these substances were procured from PubChem. Corresponding 3D target protein structures were sourced from the Protein Data Bank (http://www.rcsb.org/). The docking simulations calculated the compounds' binding free energy within the macromolecular environment. Typically, a binding energy threshold below − 1.2 kcal/mol is interpreted as a positive docking outcome, suggesting a strong likelihood of the small molecule ligand binding to the receptor.

To validate the stability of the 4-MU-FAS docking complex, MD simulations were performed using YASARA Structure 10.3.16. The system was solvated in a TIP3P water box extending 10 Å from the protein surface, with counterions added to neutralize the charge. The AMBER14 force field was applied to describe atomic interactions. Energy minimization was conducted using the steepest descent algorithm (5000 steps) to eliminate steric clashes. Subsequently, the system underwent equilibration in two phases: (1) NVT ensemble (constant particle number, volume, and temperature) at 298 K for 100 ps using the Berendsen thermostat; (2) NPT ensemble (constant pressure at 1 bar) for 200 ps with the Martyna-Tobias-Klein barostat. Production MD simulations were then carried out for 50 ns with a 2-fs integration time step under periodic boundary conditions. Trajectories were saved every 10 ps for analysis [70].

RMSD and RMSF of the protein backbone and ligand heavy atoms were calculated to assess structural convergence (RMSD < 2.0 Å) and identify dynamic binding interface residues. Binding free energy was quantified via the Molecular Mechanics/Poisson-Boltzmann Surface Area method, revealing dominant contributions from hydrophobic interactions.

For ADME prediction, the SwissADME platform (http://www.swissadme.ch/) was employed to evaluate 4-MU’s pharmacokinetic properties.

MST was utilized to explore the complex protein interactions characterized by substantial network connections. Prior to the analysis with the instrument, a concentration gradient of FAS, which was labeled using the Monolith NTTM Protein Labeling Kit RED-NHS (Catalog Number: L001), was examined, starting from a concentration of 1.89 µM and decreasing.

Molecular interaction assessments were performed using the Monolith NT 115 device and standard 4 µL glass capillaries, which were supplied by NanoTemper Technologies GmbH in Munich, Germany. The recorded fluorescence intensity from the MST experiment was employed to calculate the Kd by applying appropriate data fitting methods [71].

HepG2 cells, sourced from the Cell Bank of the Chinese Academy of Sciences, were maintained in a cell incubator under a 5% CO_2_ atmosphere at a temperature of 37 °C, utilizing a specialized HepG2 cell culture medium provided by Procell Life Science & Technology Co., Ltd. After 48 h of treatment with medium containing a mixture of 4-MU (0 μM, 25 μM, 50 μM, 75 μM, 100 μM), cell survival was assayed using the CCK-8 assay.

### In vivo anti-tumor efficacy of 4-MU

Adult male BALB/c mice, with a weight range of 18–22 g, were sourced from the Hubei Provincial Center for Disease Control and Prevention. They were accommodated in a Specific Pathogen Free environment, where temperature and humidity were meticulously regulated at 25 °C. The animals were provided with unlimited access to food and water for a week to allow them to adjust to their surroundings. The study was granted ethical approval by the Animal Ethics Committee of Hubei University, adhering to the guidelines set by the Animal Care and Use Committee of the Chinese Institute of Pharmaceutical Sciences.

Under aseptic conditions, 0.2 mL of H22 tumor cell suspension (1 × 10^6^ cells/mL) was injected into the abdominal cavity of healthy mice. After successful inoculation, the mice were monitored for abdominal tumor development. On the seventh day post-inoculation, ascites were extracted from mice with ascites tumor growth, resulting in abdominal enlargement without signs of ulceration. The ascites was centrifuged to isolate tumor cells, and the concentration was adjusted to 5 × 10^5^ cells/mL with saline. 0.2 mL of the adjusted cell suspension was injected subcutaneously into the dorsal side of 40 healthy mice under aseptic conditions. The mice were randomly divided into four groups of 10 mice each. The model group (given saline) was injected once a day, while the 4-MU treatment groups (25 mg/kg, 50 mg/kg, 100 mg/kg) were injected once a day for 14 days. Total cholesterol, triglyceride, LDL-cholesterol, and HDL-cholesterol were measured. Protein expression was determined by immunohistochemistry, and changes in tissue structure and cell morphology were observed by pathological sectioning.

### Statistical analysis

The collected experimental data were subjected to analysis using SPSS 16.0, an integrated statistical software suite, in conjunction with ImageJ, a dedicated image analysis software. The outcomes were reported as mean values complemented by their standard deviations. For evaluating the statistical significance of differences between groups, a ANOVA test was utilized. A threshold of p-value less than 0.05 was established to indicate a statistically significant disparity among the groups.

## Supplementary Information

Supplementary Material 1.

Supplementary Material 2.

## Data Availability

Data will be made available on request.
